# Delayed Dual-Energy CT (DECT) and conventional cardiac CT angiography (CCTA) in detection of chronic myocardial scar tissue: do we need delayed acquisition? Comparison with MRI

**DOI:** 10.1186/1532-429X-17-S1-P135

**Published:** 2015-02-03

**Authors:** Ekaterina Pershina, Valentin Sinitsin, Elena Mershina

**Affiliations:** Russian Federal Rehabilitation center of treatment, Moscow, Russian Federation

## Background

To compare delayed enhancement DECT with CCTA and LGE MRI for detection of scars after myocardial infarction and to analyze the possible additive value of delayed DECT as part of CCTA protocol.

## Methods

19 patients (m/f-16/3, mean age 59,6± 2,0 years) with history of myocardial infarction ( >1 year) were prospectively enrolled in the study. The CCTA protocol consisted of prospectively gated CTA and DECT. DECT was performed with single-tube 64-row CT in gemstone spectral imaging (GSI) mode with 8 min delay after contrast media injection. Using a 4-point transmurality scale CCTA images were visually assessed for first-pass arterial enhancement deficit and late enhancement in DECT images using iodine distribution maps. Per-segment analysis was performed by 2 observers independently. LGE MRI was performed after CT (range: 1-3 days) as a reference standard. Test characteristics (sensitivity and specificity, contrast ratio (CR) between normal myocardium and scar tissue) for detection of myocardial scar were calculated both for CCTA and DECT. Per segment agreement between modalities was investigated with Spearman rank correlation coefficient.

## Results

141/323 (43,7%) of LV segments showed LGE on MRI. At segmental level delayed DECT had good accuracy (90%) for scar evaluation with excellent specificity (99%) and satisfactory sensitivity (78%). CCTA protocol without integration of delayed DECT had accuracy, sensitivity and specificity 92%, 88%, 95%, respectively. Addition of delayed DECT results did not improve CCTA performance (94%, 88%, 99%, respectively). CR of scar tissue was higher for CTA 274%±29% vs. 123±6% for DECT, p=0.008). Scores of transmurality were significant lower for CCTA and delayed DECT than for MRI (p=0.015 p= 0.013, resp.).

## Conclusions

Detection of ischemic scars with delayed DECT and CCTA showed a good correlation with MRI. Delayed DECT detects myocardial scars with good accuracy but integration of delayed DECT into CCTA protocol did not improved performance of CCTA for detection of chronic scars and could be omitted from cardiac CT protocols in order to reduce radiation exposure to patient.

## Funding

N/A.

**Figure 1:**
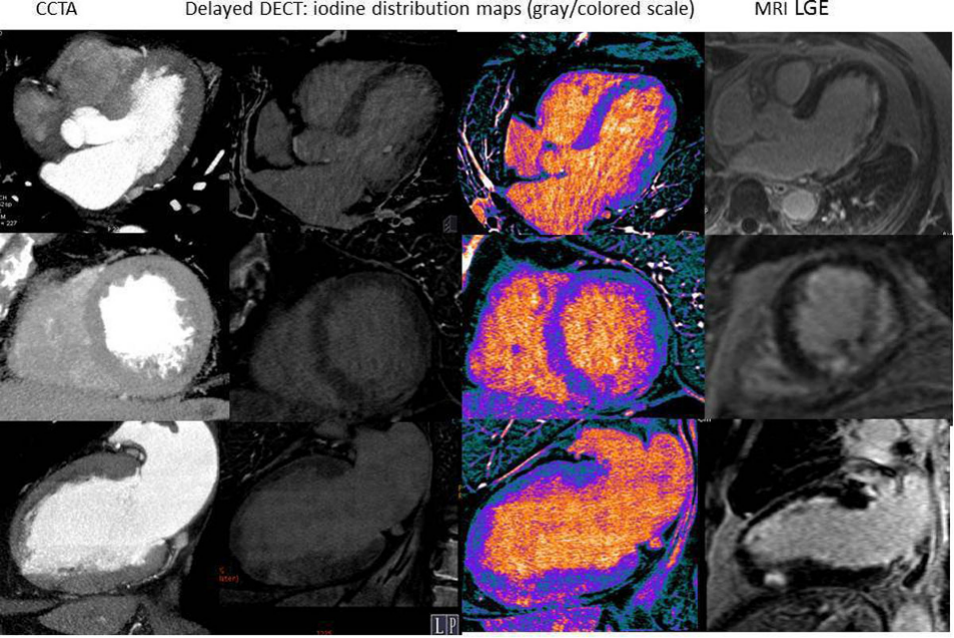
Figure 1

**Figure 2:**
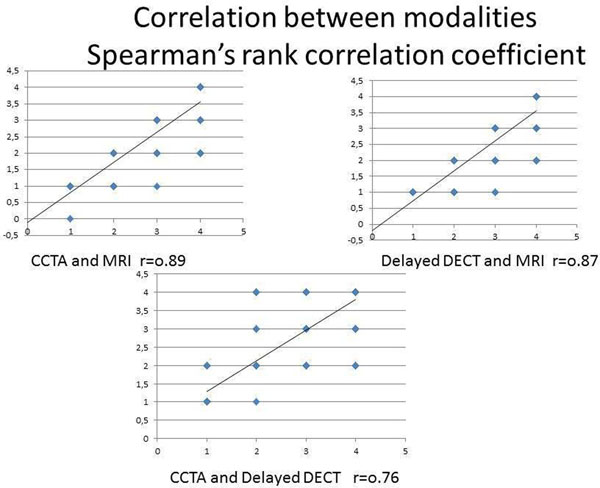
Figure 2

